# Development and outcomes of a comprehensive multidisciplinary incidental lung nodule and lung cancer screening program

**DOI:** 10.1186/s12890-020-1129-7

**Published:** 2020-04-29

**Authors:** Gregory P. LeMense, Ernest A. Waller, Cheryl Campbell, Tyler Bowen

**Affiliations:** 1 Bozeman Health Pulmonary Medicine, 937 Highland Blvd, Suite 5510, Bozeman, MT 59715 USA; 2 Blount Memorial Physicians Group, 266 Joule Street, Alcoa, TN 37701 USA

**Keywords:** Lung nodule, Lung Cancer, Incidental lung nodule, Lung Cancer screening, Interventional pulmonology

## Abstract

**Background:**

Appropriate management of lung nodules detected incidentally or through lung cancer screening can increase the rate of early-stage diagnoses and potentially improve treatment outcomes. However, the implementation and management of comprehensive lung nodule programs is challenging.

**Methods:**

This single-center, retrospective report describes the development and outcomes of a comprehensive lung nodule program at a community practice in Tennessee. Computed tomography (CT) scans potentially revealing incidental lung nodules were identified by a computerized search. Incidental or screening-identified lung nodules that were enlarging or not seen in prior scans were entered into a nodule database and guideline-based review determined whether to conduct a diagnostic intervention or radiologic follow-up. Referral rates, diagnosis methods, stage distribution, treatment modalities, and days to treatment are reported.

**Results:**

The number of patients with lung nodules referred to the program increased over 2 years, from 665 patients in Year 1 to 745 patients in Year 2. Most nodules were incidental (62–65%). Nodules identified with symptoms (15.2% in Year 1) or through screening (12.6% in Year 1) were less common. In Year 1, 27% (182/665) of nodules required a diagnostic intervention and 18% (121/665) were malignant. Most diagnostic interventions were image-guided bronchoscopy (88%) or percutaneous biopsy (9%). The proportion of Stage I-II cancer diagnoses increased from 23% prior to program implementation to 36% in Year 1 and 38% in Year 2. In screening cases, 71% of patients completed follow-up scans within 18 months. Only 2% of Year 1 patients under watchful waiting required a diagnostic intervention, of which 1% received a cancer diagnosis.

**Conclusions:**

The current study reports outcomes over the first 2 years of a lung cancer screening and incidental nodule program. The results show that the program was successful, given the appropriate level of data management and oversight. Comprehensive lung nodule programs have the potential to benefit the patient, physician, and hospital system.

## Background

Lung cancer is the leading cause of cancer-related death worldwide [1]. Age-standardized life years lost due to lung cancer is significantly worse in the southeastern United States compared to national averages [2]. While early detection markedly improves survival, only 16% of lung cancers in the United States are detected at localized stages [3]. Dedicated programs to identify and manage lung nodules may facilitate early detection and improve survival. Asymptomatic, early-stage lung nodules can be detected either as incidental nodules on thoracic computed tomography (CT) or through screening. Incidental nodules are commonly seen on thoracic and abdominal CT imaging for trauma, cardiac symptoms, or abdominal symptoms [4]. In an integrated health system report between 2006 and 2012, approximately 25–30% of all chest CT scans revealed positive findings. This corresponds to the identification of an estimated 1.57 million new lung nodules annually [5]. However, the false positive rate is high, with only about 5% of identified nodules receiving a lung cancer diagnosis within 2 years [5]. In addition, follow-up of incidentally detected nodules is generally poor [6]. In over 15,000 patients with incidental nodules from a commercial insurance database, only 36% received subsequent workup despite an estimated cost of only $1–$2 per member per year when averaged across the insured population [7]. Lung cancer screening in appropriate patients has increased early-stage detection (36–71% Stage I) [8] and reduced long-term mortality rates [9–12]. However, as with incidentally detected nodules, false positive rates are high [9]. Low-dose CT (LDCT) screening adoption has been poor despite recommendations, possibly due to lack of patient education, access to care, and reimbursement [13], particularly in the Southeastern United States [14, 15]. Screening implementation is also challenged by insufficient resources to manage positive cases according to guidelines, which is currently a requirement for reimbursement [16].

In recent years, an increasing number of centers have developed incidental lung nodule management and screening programs. Although the application of evidence-based guidelines, such as those of the Fleischner Society [17] or American College of Chest Physicians (ACCP) [18], improves follow-up rates [19], challenges in workload and workflow management, referral pathways, expertise, and systematic tracking remain barriers.

The objective of the current paper is to describe the development and results of a lung cancer screening and incidental nodule management program in a community hospital serving 5 counties in Tennessee. This paper presents the requirements for a comprehensive program, volume for both incidental nodules and screening over time, and quarterly trends in referrals, diagnostic interventions, stage distribution, treatment modalities, and financial data.

## Methods

This is a single-center, retrospective chart review. Blount Memorial Hospital is a 304-bed facility serving 5 counties in Tennessee with a 16-county pulmonary referral area. Blount Memorial Physicians Group is a multispecialty private practice with a large primary care base. Lung cancer rates in Tennessee are among the highest in the United States, with an age-adjusted rate of 76.6 new cases per 100,000 people, compared to a national average of 58 new cases per 100,000 [20].

Prior to the development of a comprehensive lung nodule clinic in 2016, the lung program at Blount Memorial Hospital consisted of a lung nodule screening service that was offered for a cash price of $99 United States dollars. A rudimentary incidental nodule program consisted of a single individual manually reviewing CT reports. There was a multidisciplinary cancer conference, but no structured program for evaluating nodules or coordinating follow-up. There was no monitoring of lung cancer screening scans, with reports only sent to the ordering physician.

Beginning in 2016, a comprehensive and coordinated lung nodule program was initiated with the aims of achieving: (1) a robust cancer screening program; (2) timely capture of incidental lung nodules; (3) cohesive care between practitioners; (4) a streamlined referral program; and (5) a multidisciplinary “virtual clinic” with a nurse navigator who coordinated care across specialties.

**Fig. 1:**
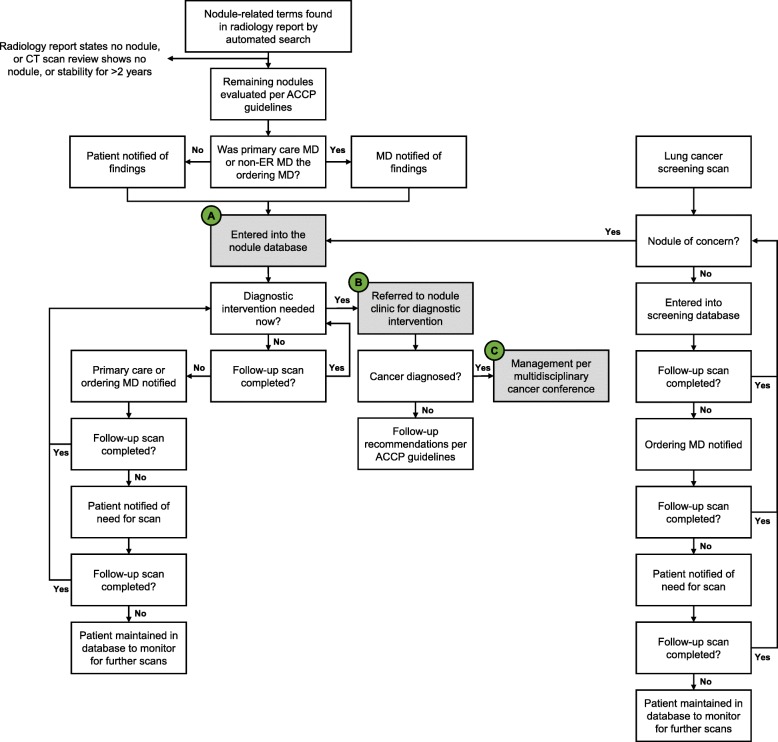
Lung nodule clinical overview. Approximately 6000 incidental nodule and screening CT scans were reviewed annually at the medical center during the study timeframe. In the first year of the program, 665 pulmonary nodules were added to the lung nodule database (“A”). Of those, 182 underwent diagnostic intervention (“B”), and 121 ultimately received a cancer diagnosis (“C”)

Figure 1 depicts the procedural flow after full program implementation. An automated electronic search was used to scan radiology dictation notes for words related to lung nodules. To ensure that no patients were missed, the entire dictation was searched (rather than only the “impression” section). Manual review of CT scans and CT scan reports identified by the electronic search then filtered out CT scans with no nodules or those with nodule stability for > 2 years. Patients entered into the lung nodule database were those with lung nodules observed to be enlarging or not seen in a prior scan. Those patients were then reviewed by two interventional pulmonologists (GPL and EAW) and evaluated based on ACCP evidence-based guidelines [21] to determine whether to conduct a diagnostic intervention or to follow with imaging.

All patients requiring diagnostic intervention (defined as invasive biopsy) were tracked. Most lung diagnostic interventions were conducted using image-guided bronchoscopy, including electromagnetic navigation bronchoscopy (ENB; superDimension™ navigation system version 7.1) and radial endobronchial ultrasound (EBUS) probe (Olympus). Mediastinal lymph node assessment was conducted using linear EBUS (Olympus BF-UC180F bronchoscope). Whenever possible, diagnosis and staging was conducted within a single procedure. Most patients were evaluated with both ENB and linear EBUS in the same procedure. Other procedures, such as CT-guided fine-needle aspiration and mediastinoscopy were only conducted when diagnosis and staging could not be accomplished with a single procedure. Thoracentesis was limited to patients with a pleural effusion of adequate size that was thought to be a malignant effusion. Symptomatic patients were defined as those with symptom(s) attributable to the lung nodule(s) at the time of presentation.

The program goal was to improve patient outcomes, with specific objectives of achieving (1) a stage shift to earlier lung cancer diagnosis and (2) treatment initiation within 30 days of first nodule detection (defined as the day of the index CT scan, or the day of the CT scan that initiated intervention for patients originally placed in observation). The program was rolled out in a stepwise fashion, with the databases started in July 2016, followed by the formal screening program on September 1, 2016 and the incidental nodule program on October 1, 2016.

Year 1 data are presented from October 1, 2016 to September 30, 2017, representing the first year of the fully operational screening and incidental management program. Available data for the “phase-in” screening period of July 2016 through September 2016 is presented separately to characterize the initial impact of screening program implementation on volume and stage shift; however, this data is more limited than the data available in Year 1 and Year 2.

Analyses were performed in Excel and data were summarized by descriptive statistics (for continuous variables) or frequencies and percentages (for categorical variables). Financial data were calculated as unbudgeted revenue generated by the nodule program, using Medicare reimbursement data for each specific year.

## Results

### Year 1 Data

**Fig. 2:**
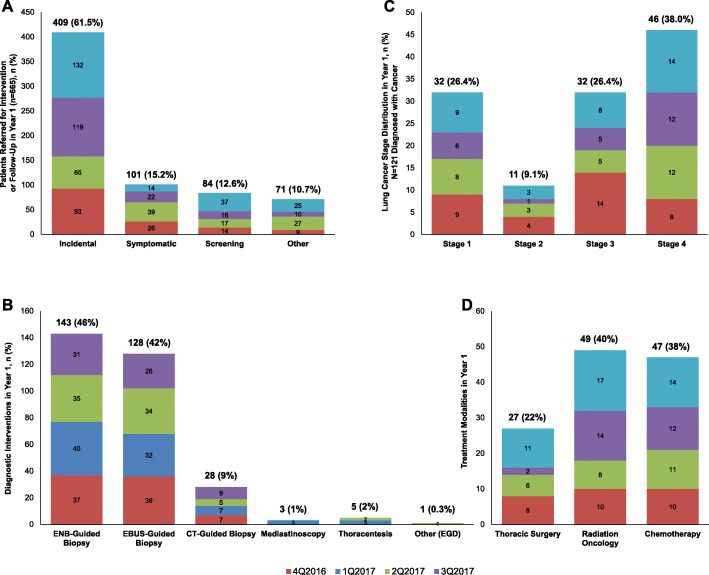
Year 1 Data. **a** Referrals to the Lung Nodule Clinic in Year 1 (*n* = 665). The majority (61.5%) of referred nodules in Year 1 were incidentally detected. Of the 255 screening scans in Year 1, 73 were referred to the lung nodule clinic for review. **b** Diagnostic Interventions in Year 1 (*n* = 182 Patients). Each patient may have more than one diagnostic procedure so individual subcategories do not sum to the total number of 182 patients. **c** Stage Distribution of Cancer Diagnoses in Year 1 of the Program. **d** Treatment Modalities in Year 1

Population-based modeling prior to program implementation predicted 1027 nodule patients in Year 1, of which 125 would require diagnostic intervention. Approximately 6000 CT scans were reviewed annually at the medical center during the study timeframe (screening and incidental). In Year 1, 1792 patients were identified by the computer search. After review of the CT report and the CT scan, 1127 were determined to not have a nodule or to have a nodule that had been stable for more than 2 years. The remaining 665 nodules were evaluated according to ACCP guidelines and entered into the database as requiring either biopsy or follow-up (Fig. 1, Box A). Of the 665 patients entered into the nodule database in Year 1, 61.5% were incidental, 15.2% were symptomatic, and 12.6% were identified through screening (Fig. 2a). During the phase-in period prior to full program implementation, 63 nodule referrals were received. The program then grew by quarter in Year 1, with 142 referrals in the first quarter of the full program, followed by 148, 167, and 208 in the subsequent three quarters.

Fifty-eight of the 665 patients were not directly referred to the lung nodule program, but were instead followed by their primary care physician or an outside pulmonologist. All of these 58 patients had small nodules that only required follow-up (watchful waiting). They were still included in the database to confirm that follow-up imaging was completed. The remaining 607 patients were managed by the nodule program.

A total of 182 patients underwent a diagnostic intervention (Fig. 1, Box B). These included classically defined lung nodules (density on imaging < 3 cm) and mass lesions and patients with concomitant airway lesions, adenopathy, pleural effusion, etc. Most diagnostic procedures (Fig. 2b) were ENB (46%) or EBUS biopsy (42%), with a relatively even distribution after the first quarter. Among the patients evaluated with ENB or EBUS, 10% underwent ENB alone, 22% underwent EBUS alone, and 68% underwent combined ENB/EBUS. In Year 1, only 7 patients (7/182; 3.8%) required a second separate procedure to obtain a diagnosis, complete staging, and/or aid in fiducial marker placement.

Of the 182 cases undergoing diagnostic intervention, 121 received a cancer diagnosis and were managed by the multidisciplinary cancer conference (Fig. 1, Box C), including 119 primary and 2 metastatic (pancreatic and renal) cancers. This represents 66.5% (121/182) of nodules undergoing diagnostic intervention and 18.2% (121/665) of all nodules in Year 1. The number of diagnostic procedures (47 vs. 44) and cancer diagnoses (35 vs. 34) remained stable between the first and last quarters of Year 1.

Early-stage cancer was detected in 35.5% (26.4% Stage 1, 9.1% Stage 2) while late-stage cancer was detected in 64.4% (26.4% Stage 3, 38.0% Stage 4; Fig. 2c). Treatment modalities (Fig. 2d) were thoracic surgery in 22%, radiation oncology in 40%, and chemotherapy in 38%.

The remaining 483 patients without diagnostic intervention were placed into “watchful waiting” (including the 58 patients not managed by the lung nodule program). After follow-up imaging, 9/483 (1.9%) required diagnostic intervention of which 5/483 (1.0%) had cancer. Three had negative biopsies and remain in follow-up with no progression and one had a pseudomonas infection that responded to treatment. A total of 474 patients remain in monitoring with no progression noted on follow-up imaging.

### Program Data through 2 years

During the second year, 745 patients were entered into the database and managed by the program, 169 (22.6%) underwent diagnostic intervention, and 86 (11.5%) received a cancer diagnosis. Four patients required a second procedure to complete staging or obtain a diagnosis. The remaining 576 patients were placed into watchful waiting. With follow-up scans, 5/576 (0.9%) required diagnostic intervention, of which 4/5 had cancer and 1/5 had a negative biopsy. A total of 572 Year 2 patients remain in monitoring.

**Fig. 3:**
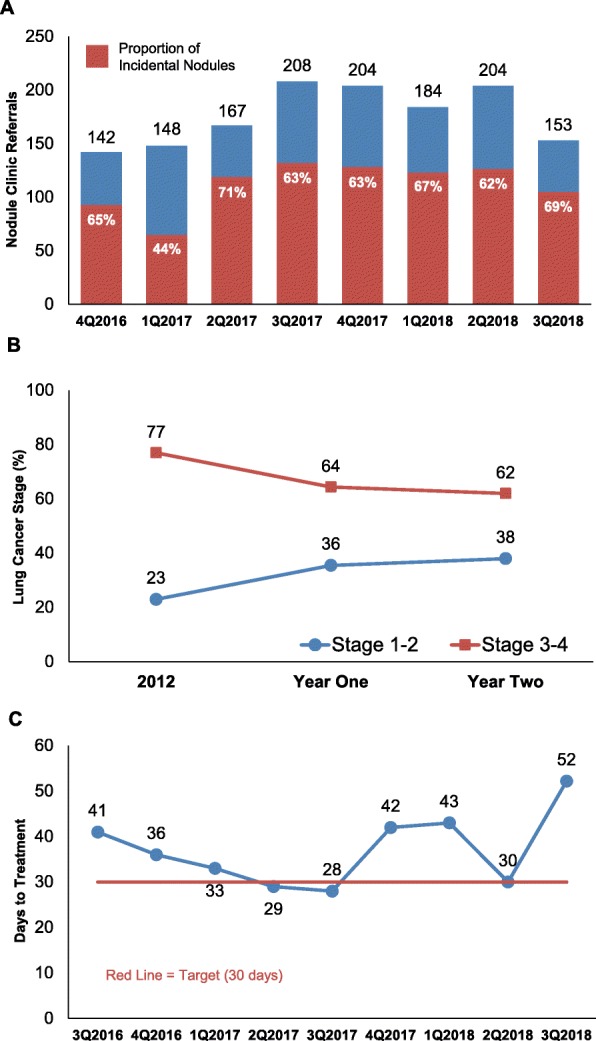
Cumulative Program Data Through 2 Years. **a** Referrals and incidental nodules by quarter over 2 years. Total referrals are shown in bars. Red portion indicates the percentage of the total represented by incidental nodules. **b** Lung cancer stage distribution at diagnosis before program implementation (2012) and in the first and second year of lung nodule program implementation. **c** Average days to treatment from first nodule detection. Red line indicates a program target objective of 30 days or fewer

Figure 3 shows cumulative data through 2 years. The number of referrals increased throughout the first 5 quarters and then levelled off slightly during Year 2 (Fig. 3a). The percentage of incidental nodules increased from 61.5% in Year 1 to 64.9% in Year 2. As shown in Fig. 3b, the relative proportion of early-stage lung cancer detection increased during the 2 years after program implementation compared to 2012 Medicare baseline data [22].

Average days to treatment over 2 years (Fig. 3c) decreased steadily in Year 1, from 41 days in the phase-in period to 36 days in the first quarter and 28 days in last quarter. An increase to 42 and 43 days was observed in the fourth quarter of 2017 through the first quarter of 2018, dropping to 30 days in the second quarter, and rising again to 52 days in the third quarter of Year 2. Potential reasons for these observations are detailed in the Discussion.

### Screening program Data

The number of new screening scans increased steadily each quarter, with 255 in Year 1 and 549 in Year 2. Patients with screening-detected nodules that required more than yearly CT follow-up were moved to the active database (29% in Year 1 and 20% in Year 2). Among first-year patients, 71% had follow-up scanning within 18 months of the initial scan.

### Financial Data

The total revenue generated by the nodule program in Year 1 was $2,193,994.81. Two-thirds of that revenue was generated by video-assisted thoracoscopic surgery, radiation oncology, and medical oncology treatment procedures, approximately 20% by imaging, and 15% by bronchoscopic or transthoracic biopsy. Revenue increased steadily through 2 years after program implementation, with $363,504 generated in the first quarter, increasing to a cumulative total of $3,812,461 at 18 months.

## Discussion

Comprehensive lung nodule programs may facilitate an earlier diagnosis and a higher cure rate. While the structure and technology exist to capture and manage patients, implementation is often challenging. This report describes the first 2 years of a lung cancer screening and incidental nodule program. Several key outcomes were observed that may be informative to other medical centers considering adoption of a comprehensive lung nodule program.

First, 62–65% of nodules in our program were incidental nodules. In Year 1, only 12.6% of nodules were identified through screening. Similarly, a report of a Northeastern United States comprehensive lung nodule program found that 80% of nodules were found incidentally and only 20% through lung cancer screening [23]. Thus, any program that does not capture incidental findings is likely to miss a significant portion of lung nodules.

Second, guideline-based interventional pulmonology review effectively reduced false positives and unnecessary invasive testing. Prior screening and incidental nodule studies have reported false positive rates as high as 96% [9, 24]. In our program, guideline-based interventional pulmonology review in Year 1 eliminated 1127 of the original 1792 CT scans identified by the radiology search to yield 665 nodules requiring intervention or radiologic follow-up. Of those, 27% (182/665) required a diagnostic intervention and 18% (121/665) received a cancer diagnosis. Only 2% of patients initially placed in “watchful waiting” during Year 1 required a diagnostic procedure after follow-up scanning, and only 1% received a cancer diagnosis. This speaks to the effectiveness of interventional pulmonologist review of CT scans based on ACCP guidelines [18].

Third, our comprehensive program increased the proportion of early stage cancer diagnoses. Interventional pulmonology programs that include advanced minimally invasive diagnostic procedures, such has ENB, are associated with increased rates of early-stage diagnosis [25–27] and fewer benign resections [25]. We observed an increased proportion of Stage I-II diagnoses in Years 1 and 2 compared to before program implementation. We did find an increase in Stage IV diagnoses at the end of Year 1, for reasons that are not entirely clear. In our practice, many Stage IV patients presenting through the emergency room had minimal or no symptoms; often, the emergency room was their first encounter with the health system. Thus, these “late presenters” may still be working through the system. Nonetheless, an overall increase in earlier stage diagnoses was observed.

Fourth, our data show that a comprehensive nodule program can increase screening rates and follow-up compliance. In our practice, almost all patients meet the United States Preventive Services Task Force (USPSTF) screening criteria [28]. While most nodules were incidental, we saw a steady increase in screening referrals over the 2 years that continues to date. This is encouraging given prior reports of poor screening adoption and compliance [29], particularly in the United States Southeast [15]. Our follow-up rate of 71% on screened patients is higher than the national average and other screening studies [19, 30], demonstrating the value of a comprehensive program to encourage compliance.

Fifth, although our analysis is limited in scope to the United States health system, our data support a meaningful financial impact for hospital and health systems of any size. We observed a total revenue of $2,193,994.81 in United States dollars in Year 1. While not all of that was “new” revenue (much of it would have been introduced into the system anyway), this number represents unbudgeted revenue generated by the nodule program. However, efficiency is also important to reduce unnecessary costs from over-management [31]. In our study, 66.5% (121/182) of nodules that underwent diagnostic intervention received a cancer diagnosis. Most of those interventions were minimally invasive ENB-guided and EBUS-guided bronchoscopy procedures. Although we did not conduct a formal cost-effectiveness analysis, our results support prior publications from Europe [32], the United Kingdom [33], Asia [34], and the United States [35, 36] demonstrating the cost-effectiveness of screening and incidental nodule programs.

Finally and most importantly, our data emphasize that careful patient navigation and oversight is essential to the success of any lung nodule program [37], especially in vulnerable populations [38]. Lung nodule management is data-intense and requires efficient and effective data oversight, as well as adequate staffing. We implemented several best practice procedures to increase patient compliance and ensure no patients were lost. As one example, our database was queried monthly to report on every patient due for a follow-up scan that month. If the patient did not return for a scan, the nurse navigator contacted the patient to schedule a follow-up. This oversight greatly increased our follow-up rates. Regular data checks are also essential to assess program efficiency and quickly address the causes of any challenges. As a second example, we had set a program objective of ≤30 days from first nodule detection to first treatment. While we achieved a steady decrease to 28 days at the end of Year 1, we observed several upticks due to program and resource challenges. In one instance, a root-cause analysis determined that the nodule program had overwhelmed the radiation oncology resources. The radiation oncology department had recently begun using a new scanner and the learning curve reduced the number of cases that could be evaluated per day. In response, we have now implemented a best practice of reviewing any cases over 40 days to treatment every quarter to identify the cause and implement process flow improvements in response. A whiteboard tracker was also established with the calculated target treatment date for every patient. Those dates were communicated to the radiation oncologist and medical oncologist offices to ensure coordinated care. These oversight best practices help ensure that temporary challenges do not become pervasive.

## Conclusions

Our experience suggests that with careful oversight, a successful lung nodule program can be implemented in any type of community health system, not just large multi-hospital systems. Our initial two-year data suggest that all three stakeholders – patients, physicians, and hospitals – can benefit from a properly run, effective program. Patients benefit from earlier diagnosis and potentially better treatment outcomes, physicians benefit from the availability of high-quality multidisciplinary care that provides a “safety net” to ensure patients aren’t lost to follow-up, and the hospital benefits from downstream revenue and keeping patients within the system. Combined, this creates a “win-win-win” situation for the healthcare system and patients as a whole.

## Data Availability

All data generated or analysed during this study are included in this published article. Additional information is available from the corresponding author on reasonable request.

## Abbreviations

ACCP: American College of Chest Physicians
CT: Computed tomography
ENB: Electromagnetic navigation bronchoscopy
LDCT: Low-dose computed tomography
USPSTF: United States Preventive Services Task Force
